# Ferroptosis in idiopathic pulmonary fibrosis: mechanisms, impact, and therapeutic opportunities

**DOI:** 10.3389/fimmu.2025.1567994

**Published:** 2025-05-21

**Authors:** Mingjun Yao, Zheng Liu, Wei Zhao, Siyuan Song, Xiaobo Huang, Yi Wang

**Affiliations:** ^1^ School of Medicine, University of Electronic Science and Technology of China, Chengdu, China; ^2^ Pathology Department, University of Texas, MD Anderson Cancer Center, Houston, TX, United States; ^3^ Department of Neuroscience, Baylor College of Medicine, Houston, TX, United States; ^4^ Department of Critical Care Medicine, Sichuan Provincial People’s Hospital, University of Electronic Science and Technology of China, Chengdu, Sichuan, China; ^5^ Translational Clinical Immunology Medicine Key Laboratory of Sichuan Province, Center of Organ Transplantation, Sichuan Academy of Medical Sciences and Sichuan Provincial People's Hospital, Chengdu, Sichuan, China

**Keywords:** ferroptosis, immune homeostasis, idiopathic pulmonary fibrosis (IPF), type I interferon, interleukin, tumor necrosis factor, transforming growth factor, toll-like receptor

## Abstract

Idiopathic pulmonary fibrosis (IPF) is a fatal interstitial lung disease characterized by progressive scarring, alveolar destruction, and limited therapeutic options. Although the exact etiology of IPF remains unclear, emerging evidence suggests that ferroptosis, an iron-dependent form of regulated cell death driven by lipid peroxidation and oxidative stress, plays a significant role in its pathogenesis. Ferroptotic stress not only compromises alveolar epithelial cell integrity, but also triggers inflammatory responses and profibrotic signaling cascades that activate and sustain fibroblast dysfunction. This review delineates the core regulatory pathways of ferroptosis, iron metabolism, lipid peroxidation, antioxidant defenses, mitochondrial remodeling, and RNA editing, with an emphasis on their relevance in IPF. We explore how epithelial injury and macrophage-derived signals initiate ferroptosis, and how fibroblast subsets, shaped by scRNA-seq-defined heterogeneity and plasticity, respond to these cues by reinforcing ECM deposition and oxidative stress. Therapeutic avenues targeting ferroptosis, including antioxidant supplementation, iron chelation, and modulation of lipid metabolism, are discussed alongside cell-specific interventions and nanodelivery strategies. By integrating recent advances in molecular profiling and ferroptosis biology, this review provides a framework for leveraging ferroptosis as a tractable target in IPF and identifies novel directions for precision antifibrotic therapy.

## 1 Introduction

### 1.1 Overview of pulmonary fibrosis

Pulmonary fibrosis (PF) encompasses a group of chronic, progressive lung diseases characterized by excessive extracellular matrix (ECM) deposition, tissue remodeling, and loss of lung elasticity. These pathological changes impair gas exchange and ultimately lead to respiratory failure, with advanced cases often necessitating lung transplantation (1). The development of PF involves a complex interplay of genetic predisposition, environmental exposures, and dysregulated tissue repair. Repeated alveolar epithelial injury, unresolved inflammation, and aberrant fibroblast activation drive progressive scarring, as activated fibroblasts secrete ECM components that perpetuate fibrosis (2). Despite significant advances in understanding the molecular mechanisms underlying PF, effective treatment options remain limited, underscoring the need for continued research.

### 1.2 Idiopathic pulmonary fibrosis

Idiopathic pulmonary fibrosis (IPF), the most severe and common subtype of PF, affects approximately 25,000 new individuals annually in the United States. Histologically, IPF is defined by the usual interstitial pneumonia (UIP) pattern, characterized by patchy fibrosis, fibroblastic foci, and honeycombing (3). Unlike other interstitial lung diseases, IPF follows a particularly aggressive clinical course, with a median survival of only 3–5 years after diagnosis (4).Current antifibrotic therapies, including pirfenidone and nintedanib, may slow disease progression but fail to halt or reverse fibrosis, highlighting the urgent need for more effective treatments (5).

### 1.3 Ferroptosis and IPF

Ferroptosis, a unique form of cell death derived by iron overload and lipid peroxidation, has emerged as a critical contributor to oxidative stress and chronic inflammation in IPF. This process disrupts alveolar epithelial integrity, activating profibrotic signaling pathways, including fibroblasts proliferation, and ECM deposition—hallmarks of IPF pathogenesis (6). Unlike apoptosis, ferroptosis is non-apoptotic and pro-inflammatory, amplifying tissue injury and perpetuating fibrotic remodeling. As such, targeting ferroptosis represents a promising therapeutic strategy (7).

This review aims to synthesize emerging insights into the mechanistic role of ferroptosis in IPF, with a particular focus on how ferroptotic stress influences fibroblast responses, epithelial-fibroblast crosstalk, and fibrotic progression. We highlight recent findings on the cellular and molecular mechanisms driving ferroptosis, its impact on tissue remodeling, and potential therapeutic opportunities. By examining both epithelial and stromal contributions to ferroptosis-driven pathology, we aim to provide a framework for identifying novel targets for disease interception and treatment.

## 2 Ferroptosis: core mechanisms and regulatory pathways

Ferroptosis is a distinct form of regulated cell death that differs fundamentally from apoptosis, necroptosis, and pyroptosis. Unlike these pathways, which involve caspase activation, membrane pore formation, or DNA fragmentation, ferroptosis is driven by iron-catalyzed lipid peroxidation and oxidative damage to cellular membranes. Its regulation centers on four interconnected processes: iron metabolism, polyunsaturated fatty acids (PUFA) peroxidation, antioxidant defense failure, and mitochondrial dysfunction (8).

### 2.1 Iron metabolism and ferroptosis

Iron is central to ferroptosis, as its redox activity catalyzes the formation of reactive oxygen species (ROS) via Fenton reactions, triggering cellular membranes damage and cell death (9). Cellular iron homeostasis is maintained by tightly regulated pathways involving iron import (e.g., transferrin receptor 1 [TfR1]), export (e.g., ferroportin), and storage (e.g., ferritin) (10–12). Disruption of this balance leads to labile iron accumulation, sensitizing cells to ferroptosis (13).

A critical regulator of intracellular iron levels is ferritinophagy, the selective autophagic degradation of ferritin mediated by nuclear receptor coactivator 4 (NCOA4). This process releases stored iron into the cytosol, increasing susceptibility to ferroptosis (14). Recent studies have shown that enhanced ferritinophagy in stressed alveolar epithelial cells contributes to iron overload and ferroptotic injury in pulmonary fibrosis models (7).

In the context of IPF, elevated pulmonary iron levels and altered expression of iron-handling proteins have been observed in patient lungs, particularly in fibrotic regions (6). Experimental inhibition of iron accumulation or ferritinophagy using deferoxamine or NCOA4 knockdown attenuates epithelial cell death and collagen deposition in bleomycin-induced fibrosis models, supporting the pathogenic role of iron dysregulation in IPF (15).

Together, these findings underscore aberrant ion metabolism, notably excessive import, ferritin degradation, and impaired export, as a key initiator of ferroptosis in IPF and related fibrotic lung diseases. Targeting these dynamics may attenuate the epithelial damage and profibrotic microenvironment characteristic of IPF.

### 2.2 Lipid peroxidation and PUFA susceptibility

Lipid peroxidation of polyunsaturated fatty acids (PUFAs), particularly within phospholipid membranes, is a defining feature of ferroptosis (16). This process is driven by the enzymatic and non-enzymatic oxidation of PUFA-containing phosphatidylethanolamines (PUFA-PEs), such as those incorporating arachidonic acid (AA) and adrenic acid (AdA). Among these, AdA-phosphatidylethanolamine (AdA-PE) has emerged as a critical pro-ferroptotic lipid species, whose oxidation initiates membrane damage and cell death.

Key enzymes regulate the synthesis and incorporation of PUFAs into membrane phospholipids. Acyl-CoA synthetase long-chain family member 4 (ACSL4) activates arachidonic acid (AA) and adrenic acid (AdA) to form PUFA-CoAs, which are subsequently esterified into phospholipids by Lysophosphatidylcholine acyltransferase 3 (LPCAT3), yielding PUFA-PEs such as AdA-PE (17, 18). These PUFA-PEs are highly prone to peroxidation, especially under oxidative stress. Lipoxygenases (LOXs), such as ALOX15, enzymatically oxidize these substrates, producing lipid hydroperoxides (PE-OOH) that execute ferroptosis (19, 20).

In the setting of IPF, elevated ACSL4 and LOXs expression has been documented in fibrotic lung tissue and alveolar epithelial cell, correlating with enhanced lipid peroxidation and fibrotic progression (21–23). Genetic or pharmacological inhibition of ACSL4 or LOXs reduces lipid peroxidation, preserves epithelial cell integrity, and mitigates fibrotic remodeling (21, 24, 25). Furthermore, LPCAT3 deficiency confers partial resistance to ferroptosis by reducing PUFA incorporation into membranes, highlighting its potential as a regulatory checkpoint and therapeutic target (26).

These findings underscore the significance of PUFA metabolism and lipid peroxidation in ferroptosis execution and IPF pathogenesis. Targeting these upstream processes may attenuate ferroptotic epithelial damage and downstream fibroblast activation.

### 2.3 Antioxidant defense

**Figure 1:**
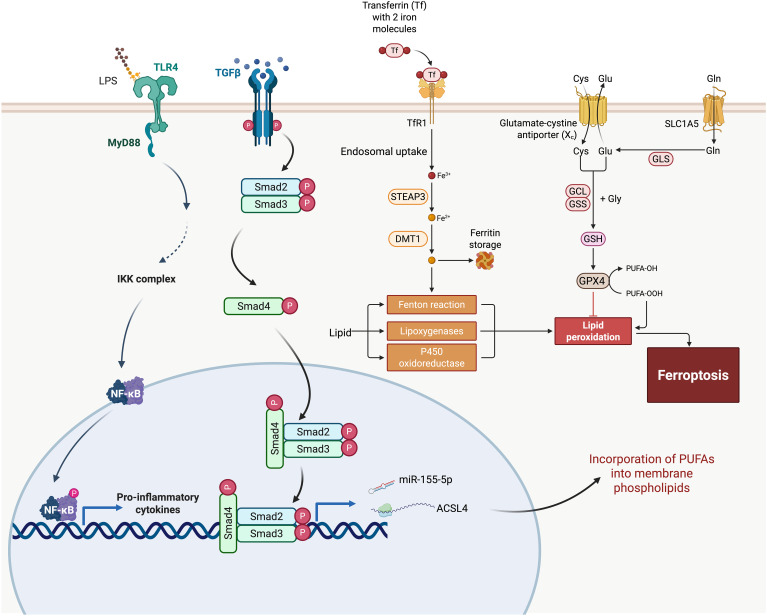
Integration of ferroptosis pathways with fibrogenic signaling in idiopathic pulmonary fibrosis (IPF). This schematic outlines the molecular crosstalk between ferroptosis and pro-fibrotic pathways in IPF. Iron uptake through transferrin receptor 1 (TfR1) and reduction by STEAP3 and DMT1 leads to intracellular Fe²⁺ accumulation, which catalyzes ROS production via the Fenton reaction. Oxidative lipid metabolism mediated by lipoxygenases and P450 oxidoreductase contributes to lipid peroxidation, a key step in ferroptosis. Glutathione (GSH) synthesis and GPX4 activity act as antioxidant defenses against lipid peroxides, while ACSL4 facilitates the incorporation of polyunsaturated fatty acids (PUFAs) into membrane phospholipids, increasing vulnerability to peroxidation. The figure also illustrates how ferroptosis intersects with the TGF-β/Smad and TLR4/NF-κB pathways—major drivers of fibrosis. TGF-β signaling activates Smad2/3/4 complexes, leading to transcriptional induction of fibrosis-associated genes. Concurrently, TLR4 signaling via MyD88 activates NF-κB, promoting the expression of pro-inflammatory cytokines. These pathways may be further amplified by ferroptotic cell death and ROS, exacerbating tissue damage and fibrotic remodeling. miR-155-5p and other regulators are implicated in enhancing ACSL4 expression, linking immune signaling to ferroptotic susceptibility. Together, this figure illustrates a feedforward loop between ferroptosis and fibrogenesis in IPF.

Oxidative lipid damage is a hallmark of ferroptosis, counteracted by a network of antioxidant systems that preserve membrane integrity and cell survival. Among these, the glutathione peroxidase 4 (GPX4) pathway serves as the central defense mechanism. GPX4 utilizes reduced glutathione (GSH) to neutralize lipid hydroperoxides (PUFA-PE-OOH) into non-toxic lipid alcohols, thereby preventing ferroptotic cell death (**Figure 1**) (27). In IPF, both GPX4 activity and intracellular GSH levels are significantly diminished in injured alveolar epithelial cells, linking ferroptosis to epithelial dysfunction and fibrosis (28).

Beyond GPX4, alternative systems also protect cells from lipid peroxidation. Ferroptosis suppressor protein 1 (FSP1) functions independently of GSH/GPX4 by reducing coenzyme Q10 (CoQ10) to its antioxidant form, CoQH_2_, which directly quenches lipid radicals in the plasma membrane (29). Activation of FSP1 has shown protective effects against ferroptosis in non-pulmonary systems, and emerging data suggest that this axis may also safeguard lung epithelium, though its role in IPF warrants further exploration (30–35).

Another key layer of defense is governed by the transcription factor nuclear factor erythroid 2–related factor 2 (NRF2). Upon oxidative stress, NRF2 translocates to the nucleus and induces expression of genes involved in GSH synthesis (e.g., GCLC, GCLM) (36), iron sequestration (e.g., FTH1), and detoxification enzymes (37). In pulmonary fibrosis models, NRF2 deficiency exacerbates lung injury and inflammation (38), while pharmacological activation of NRF2 restores redox balance, limits ferroptotic damage, and ameliorates fibrotic remodeling (37, 37).

Additionally, the GTP cyclohydrolase-1 (GCH1)/tetrahydrobiopterin (BH4) axis has recently been identified as a novel ferroptosis defense mechanism. GCH1 catalyzes the synthesis of BH4, a potent radical-trapping antioxidant that also preserves CoQ10 levels and stabilizes lipid membranes (39, 40). BH4 supplementation or GCH1 overexpression has been shown to suppress ferroptosis in oxidative injury models and may represent a therapeutic option for fibrotic lung conditions (39, 41).

Collectively, these antioxidant mechanisms—GPX4/GSH, FSP1/CoQ10, NRF2 signaling, and GCH1/BH4—interact to mitigate ferroptotic damage in epithelial cells. In the setting of IPF, where these defenses are often compromised, restoring or enhancing antioxidant capacity may not only protect alveolar integrity but also interrupt the feedback loop of oxidative stress and fibroblast activation that drives fibrosis.

### 2.4 Mitochondrial involvement and metabolic rewriting

Mitochondria serve as key modulators of ferroptosis sensitivity through their roles in reactive oxygen species (ROS) production, metabolic flux, and the maintenance of iron–sulfur (Fe–S) clusters (42). Under physiological conditions, mitochondrial respiration tightly regulates ROS levels. However, in the fibrotic lung microenvironment, persistent epithelial stress and mitochondrial dysfunction promote excessive mitochondrial ROS (mtROS), which synergize with cytosolic lipid peroxidation to drive ferroptotic cell death (43).

In IPF, alveolar epithelial cells exhibit disrupted mitochondrial dynamics and defective oxidative phosphorylation, which contribute to elevated mtROS production (44). These ROS not only damage mitochondrial membranes but also amplify lipid peroxidation cascades initiated in the cytoplasm, sensitizing cells to ferroptosis (45). Targeting mtROS with agents such as MitoQ have been shown to attenuate ferroptosis and fibrosis in preclinical models by restoring mitochondrial function and reducing lipid ROS (46).

Additionally, mitochondria are crucial for the synthesis and maintenance of Fe–S clusters, which are essential cofactors for numerous redox enzymes. Loss of Fe–S cluster stability, often observed under oxidative stress, leads to the release of free iron into the mitochondrial matrix, exacerbating Fenton chemistry and fueling ferroptosis (42). This mitochondrial iron dysregulation creates a feed-forward loop of oxidative injury that accelerates epithelial dysfunction and fibrosis.

These findings highlight the pivotal role of mitochondrial dysfunction in ferroptosis-driven fibrosis. Interventions aimed at restoring mitochondrial health represent a promising strategy to break the cycle of epithelial injury and fibrogenesis.

### 2.5 Potential biomarkers of ferroptosis

Translating ferroptosis from a mechanistic concept into a clinical tool for IPF will require reliable, minimally invasive biomarkers that reflect iron overload, lipid peroxidation, and antioxidant failure in the lung.

#### 2.5.1 Lipid−peroxidation byproducts

Quantification of malondialdehyde (MDA) and 4−hydroxynonenal (4−HNE) in bronchoalveolar lavage fluid (BALF) or exhaled breath condensate provides a direct readout of membrane lipid damage (47). Elevated MDA and 4−HNE levels correlate with disease severity and have been reported in fibrotic lung models (48).

#### 2.5.2 Enzymatic and protein markers

GPX4 activity or expression can be measured by ELISA or immunohistochemistry on BALF cells or transbronchial biopsies; reduced GPX4 is a hallmark of ferroptotic susceptibility (49).

ACSL4 and LOX family members are often upregulated in IPF lung tissue; their mRNA or protein levels may serve as positive indicators of ongoing lipid remodeling and peroxidation (50).

#### 2.5.3 Iron−handling molecules

Ferritin, transferrin saturation, and labile iron pool measurements in serum or BALF reflect systemic and alveolar iron loading (51).

NCOA4 expression in airway epithelial cells—assessed by qPCR—may indicate enhanced ferritinophagy and intracellular iron release (52).

#### 2.5.4 Ferroptosis−related gene signatures

Transcriptomic analyses of IPF BALF have identified an eight−gene ferroptosis signature (NRAS, EMP1, SLC40A1, MYC, ANGPTL4, PRKCA, MUC1, GABARAPL1) that distinguishes IPF patients from healthy controls and associates with prognosis. In murine bleomycin models, 20 additional ferroptosis−linked genes (e.g., ALOX15, CDO1, JUN, SLC2A1, GPX2) were differentially expressed in fibrotic versus normal lung (53).

Taken together, these biomarkers capture complementary facets of ferroptosis—upstream iron dysregulation, core lipid peroxidation events, failed antioxidant defenses, and downstream gene expression changes. Future efforts should standardize assay techniques, validate candidate markers in large IPF cohorts, and develop multi−analyte panels that maximize sensitivity and specificity for early detection, patient stratification, and monitoring of ferroptosis−targeted therapies.

## 3 Ferroptosis in the development of IPF

### 3.1 Environmental and intrinsic triggers

Alveolar epithelial cells (AECs), particularly type II alveolar epithelial cells (AECIIs), are central to the initiation and progression of IPF due to their roles in maintaining alveolar integrity, surfactant production, and epithelial regeneration (54–56). A growing body of evidence indicates that these cells exhibit heightened susceptibility to ferroptosis under stress conditions, rendering them a critical cellular trigger for fibrotic remodeling in the lung (57). In lungs of IPF patients, AECIIs often show impaired antioxidant capacity and evidence of accumulated oxidative damage, correlating with increased cell death and tissue injury (58). Unlike fibroblasts or immune cells, AECIIs reside at the frontline of the alveolar surface and are continually exposed to environmental, mechanical, and metabolic stressors, which may predispose them to lipid peroxidation and iron-dependent cell death (56).

Multiple environmental and intrinsic factors converge to induce ferroptosis in AECIIs, contributing to the initiation and progression of IPF. Environmental insults such as airborne pollutants, cigarette smoke, and occupational exposures (e.g., asbestos, silica) are well-documented oxidative stress in the lung. These insults generate ROS that overwhelm cellular antioxidant defenses, promote lipid peroxidation, and drive ferroptotic cell death in AECs (56, 58). Additionally chronic or recurrent respiratory infections, such as those caused by herpesviruses and bacterial colonization, can act as exogenous triggers of epithelial stress and ferroptosis (59, 60).

Intrinsic factors, including aging and genetic predisposition, further sensitize lung epithelial cells to ferroptosis (61). Age-associated mitochondrial dysfunction, telomere shortening, and impaired antioxidant responses all contribute to a ferroptosis-permissive environment (62). Genetic mutations in surfactant protein C (SFTPC) or telomerase components (TERT, TERC) compromise proteostasis and telomere maintenance, increasing epithelial susceptibility to oxidative damage and Ferroptosis (63, 64).

These environmental and intrinsic triggers for ferroptosis are also reported causes of IPF (65–68). Additionally, they may act in concert with key fibrotic signaling pathways—notably the TGF-β/Smad and TLR4/NF-κB axes—to drive chronic epithelial injury and fibrotic remodeling.

### 3.2 TGF-β signaling pathway

TGF-β is a central mediator of fibrotic responses and remains persistently activated in the lungs of patients with IPF (69). Its activation is closely linked to both environmental and intrinsic stimuli that disrupt cellular homeostasis and provoke oxidative stress. Once activated, TGF-β binds to its receptor complex, initiating phosphorylation of Smad2 and Smad3, which then form a complex with Smad4 and translocate to the nucleus and regulate gene transcription (70). In AECs, this cascade not only promotes epithelial–mesenchymal transition (EMT) and cell cycle arrest, but also contributes to a pro-ferroptotic intracellular environment. Specifically, it upregulate pro-ferroptotic enzymes, such as ACSL4, increases ROS production, and suppresses antioxidant defense mechanisms—thereby facilitating lipid peroxidation and ferroptotic cell death (**Figure 1**) (7, 71).

Beyond transcriptional regulation, TGF-β signaling modulates ferroptosis through its interplay with microRNAs (miRNAs)—key post-transcriptional regulators implicated in IPF pathogenesis (72). TGF-β can both induce and be modulated by specific miRNAs, forming a feedback network that shapes epithelial stress responses (72, 73). For example, Kong et al. demonstrated that TGF-β/Smad4 signaling upregulates miR-155, which in turn promotes TGF-β-induced epithelial–mesenchymal transition (EMT), tight junction dissolution, and cell migration (74). In bleomycin-induced pulmonary fibrosis models, inhibition of miR-155-5p reduced expression of TGF-β1, IL-1β, and TNF-α, attenuating inflammatory and fibrotic progression (**Figure 1**) (75). Given miR-155’s reported role in enhancing oxidative stress and mitochondrial dysfunction, its upregulation may also sensitize epithelial cells to ferroptosis. More broadly, TGF-β-responsive miRNAs may act as intermediaries that promote ferroptotic cell death by suppressing ferroptosis-inhibitory targets (e.g., GPX4, SLC7A11) or amplifying pro-oxidative signaling (76–78). Thus, miRNA dysregulation represents a layer of ferroptosis control embedded within canonical TGF-β signaling, contributing to the chronic epithelial injury and maladaptive repair that characterize IPF.

Moreover, TGF-β has been shown to epigenetically repress ferroptosis-protective genes (79). In models of pulmonary fibrosis, TGF-β signaling increases the expression of methylation regulators (e.g., UHRF1), which silence GPX4 and FSP1, two key suppressors of ferroptosis (30). As a result, AECs become highly susceptible to lipid ROS accumulation and ferroptotic cell death.

The ferroptotic loss of AECs is a critical pathological event that precedes and perpetuates fibrotic remodeling. Dead or dying epithelial cells release damage-associated molecular patterns (DAMPs), stimulate the recruitment of profibrotic macrophages, and enhance TGF-β production in a self-amplifying loop (80). Additionally, the loss of epithelial-derived niche factors (e.g., WNT ligands, BMPs) destabilizes the epithelial-mesenchymal balance, promoting fibroblast activation and extracellular matrix (ECM) deposition (81). In this context, TGF-β/Smad signaling serves as both a driver and amplifier of ferroptosis-induced epithelial damage, establishing a mechanistic link between chronic injury, ferroptotic stress, and irreversible fibrotic progression in IPF (82, 83).

### 3.3 NF-κB signaling pathway

The NF-κB signaling pathway plays a pivotal role in connecting innate immune activation to ferroptotic cell death and fibrotic remodeling in the lung (84). Environmental insults such as cigarette smoke or bacterial endotoxins (e.g., LPS), as well as intrinsic triggers including DAMPs released from stressed or dying epithelial cells, can engage TLR4 receptors on AECs (85). Upon activation, TLR4 signals through MyD88-dependent pathways to induce NF-κB nuclear translocation, leading to transcription of pro-inflammatory cytokines (e.g., IL-6, TNF-α) and enzymes involved in iron metabolism and lipid peroxidation, both key components of ferroptosis (**Figure 1**) (86). In LPS-induced acute lung injury models, inhibition of TLR4/NF-κB signaling has been shown to alleviate ferroptosis and reduce pulmonary damage (84).

In AECs, TLR4/NF-κB activation contributes to a pro-ferroptotic intracellular environment by upregulating genes such as NOX1 and NOX4 (promoting ROS production) and ACSL4 (increasing PUFA-containing phospholipid substrates), while suppressing ferroptosis-protective genes like GPX4 through redox imbalance and inflammatory stress (86–88). Sustained NF-κB signaling also impairs the cellular antioxidant response by disrupting Nrf2 activity, further sensitizing AECs to ferroptosis (89).

Notably, there is growing evidence of functional crosstalk between TLR4/NF-κB and TGF-β/Smad signaling in fibrotic lung disease (89). For instance, NF-κB activation has been shown to enhance TGF-β expression and facilitate its autocrine and paracrine amplification, while TGF-β signaling can stabilize TLR4 expression and amplify NF-κB-driven transcriptional responses, creating a positive feedback loop that amplifies inflammatory and fibrotic responses (86, 90, 91). Together, these pathways synergistically promote AEC ferroptosis, inflammation, and the activation of fibroblasts and myofibroblasts, ultimately accelerating ECM deposition and lung architecture distortion.

Through this integrative mechanism, TLR4/NF-κB acts not only as a sensor of epithelial injury but as an amplifier of ferroptosis-associated inflammation, bridging environmental triggers with immune activation and fibrotic remodeling—hallmarks of IPF.

## 4 Ferroptosis and IPF progression

### 4.1 Inflammatory amplification

Ferroptosis contributes not only to the initiation of IPF, but also to its chronic progression through sustained inflammatory amplification by perpetuating a cycle of epithelial injury, immune activation, and fibrotic remodeling (92).

One critical consequence of ferroptosis in AECs is the release of DAMPs, including high-mobility group box 1 (HMGB1), extracellular ATP, and oxidized phospholipids (oxPLs), which act as endogenous triggers of innate immune responses (93, 94). These DAMPs activate pattern recognition receptors (PRRs), notably TLR4 and RAGE (receptor for advanced glycation end products) on neighboring epithelial cells, infiltrating neutrophils, macrophages, and resident fibroblasts (95, 96). This activation triggers the NF-κB signaling cascade, leading to a transcriptional upregulation of pro-inflammatory cytokines (e.g., TNF-α, IL-1β, IL-6), chemokines, and adhesion molecules, which in turn recruit more immune cells to the injury site, and driving chronic inflammation and tissue damage (85).

Additionally, ferroptosis-derived oxidized lipid mediators, such as 4-HNE and MDA, can further potentiate inflammation by inducing oxidative stress and enhancing NF-κB signaling in a feed-forward loop (97–99). These lipid peroxidation products not only propagate tissue injury but also impair anti-inflammatory resolution processes (100).

Moreover, the influx of neutrophils and pro-inflammatory macrophages contributes to the formation of a self-amplifying inflammatory circuit. Neutrophils release myeloperoxidase (MPO) and ROS, exacerbating oxidative stress (101), while M1-like macrophages secrete further pro-inflammatory cytokines, establishing a chronic inflammatory niche (102). This chronicity hinders epithelial repair and sets the stage for fibrogenic signaling cascades.

Taken together, ferroptosis-induced DAMP release, cytokine production, and ROS generation converge to establish a vicious cycle of oxidative stress and inflammation, which not only amplifies immune activation but also primes the lung microenvironment for fibrotic transformation.

### 4.2 Macrophage polarization

Ferroptosis profoundly influences the immune landscape in the fibrotic lung, particularly by modulating macrophage recruitment and polarization—key processes that drive the transition from inflammation to fibrosis in IPF (86, 102). Ferroptotic AECs release bioactive molecules, including DAMPs, oxPLs, and lipid aldehydes (e.g.,4-HNE, MDA), promote monocyte infiltration by triggering the release of chemokines (like CCL2) and cytokines. These signals guide the infiltration of circulating monocytes into the injured alveolar niche and promoting their differentiation into macrophages (103). Among the heterogenous macrophage populations in the lung, monocytes-induced alveolar macrophage (Mo-AMs) have emerged as key fibrogenic players. Unlike tissue-resident alveolar macrophages, Mo-AMs expand during injury and persist long-term in the fibrotic lung. Genetic ablation studies have shown that depletion of Mo-AMs—but not embryonically derived resident alveolar macrophages—significantly ameliorates asbestos-induced pulmonary fibrosis (104). This persistence and functional specialization of Mo-AMs underscores their central role in driving sustained inflammation, secreting profibrotic mediators like TGF-β, IL-10, and CCL18, and orchestrating fibroblast activation and ECM deposition (103, 105).

Once recruited into the injured alveolar niche, infiltrating monocytes differentiate into macrophages under the influence of the ferroptotic microenvironment. This niche is enriched with DAMPs, oxPLs, and lipid peroxidation products, which collectively shape the phenotypic fate of these newly differentiated macrophages. Initially, exposure to DAMPs and ROS may transiently promote M1-like polarization characterized by pro-inflammatory responses. However, as the injury becomes chronic, persistent exposure to immunomodulatory signals—including IL-10, TGF-β, oxidized phosphatidylethanolamines (oxPEs), and lipid aldehydes like 4-HNE—progressively skews macrophages toward a profibrotic M2-like phenotype (106). Moreover, certain ferroptotic signaling products actively reinforce this polarization: oxidized phospholipids serve as ligands for PPARγ, a nuclear receptor that stabilizes M2 polarization while suppressing M1-like inflammatory gene expression (107, 108).

These M2-like macrophages become central orchestrators of fibrosis progression. They secrete a spectrum of profibrotic mediators, including TGF-β1, IL-10, arginase-1, and CCL18, which promote fibroblast proliferation, myofibroblast differentiation, and excessive ECM deposition (109). Among these, CCL18 is particularly notable for its strong correlation with disease severity in IPF patients (110).

In sum, ferroptosis not only recruits macrophages to sites of injury but also reprograms their phenotype, transforming them from inflammatory responders into active participants in fibrotic remodeling. This dual role makes macrophage polarization a crucial node in the ferroptosis-IPF axis and a promising target for therapeutic intervention.

### 4.3 Fibroblast activation and differentiation

Ferroptosis-induced loss of AECs disrupts the epithelial–mesenchymal equilibrium that is critical for maintaining pulmonary homeostasis. The depletion eliminates key antifibrotic signals—such as bone morphogenetic proteins (BMPs) and WNT pathway inhibitors—leading to the release of fibroblasts from their quiescent state and driving their proliferation and excessive collagen deposition (111, 112). The resulting imbalance forsters the expansion of fibroblastic foci, the pathological hallmark of progressive IPF, which are associated with poor clinical outcomes (113).

Fibroblasts generally exhibit resistant to ferroptosis, owning to their elevated antioxidant defenses (114). However, they are highly sensitive to ferroptotic signals released from adjacent epithelial cells. DAMPs, pro-inflammatory cytokines (e.g., IL-6, IL-1β), and lipid peroxidation products released during ferroptosis can activate fibroblasts and induce their differentiation into α-smooth muscle actin (α-SMA)-expressing myofibroblasts (115). Notably, oxPLs can directly engage TLR4 on fibroblasts, initiating downstream TGF-β and NF-κB signaling pathways that reinforce fibroblast activation and survival (116).

Differentiation into α-SMA^+^ myofibroblasts is a pivotal event in IPF progression. α-SMA expression confers contractile properties that enable myofibroblasts to remodel the ECM, contributing to increased tissue stiffness, impaired gas exchange, and further activation of latent TGF-β, thus perpetuating the fibrotic loop (117, 118). These myofibroblasts are the primary producers of ECM proteins, secreting abundant type I/III collagen, fibronectin, and tenascin-C, leading to progressive matrix deposition and scarring (119).

Crucially, myofibroblasts exhibit resistance to apoptosis, allowing them to persist even after the initial injury has resolved, sustaining the chronicity of IPF (120). They also secrete profibrotic cytokines such as TGF-β and IL-6, which promote further fibroblast differentiation and recruit additional inflammatory and fibrogenic cells, thereby amplifying a positive feedback loop that sustains fibrosis (121–123). Ferroptotic AECs further exacerbate this process by releasing matricellular proteins such as periostin, which stimulate fibroblast proliferation and ECM deposition within fibrotic niches (124).

Recent studies highlight that fibroblast activation is not a uniform process. Single-cell transcriptomic profiling has revealed marked heterogeneity among alveolar fibroblast subpopulations, including age-dependent subsets with distinct sensitivity to oxidative stress and ferroptotic signals (125). These subpopulations follow different differentiation trajectories—some favoring fibrosis progression while others may contribute to resolution—thus influencing the spatial and temporal heterogeneity of fibrotic remodeling. Ferroptosis likely exerts differential effects on these subsets, selectively promoting the expansion of profibrotic myofibroblast lineages while sparing or even suppressing anti-fibrotic populations (125).

This heterogeneity is further reflected in the characteristic patchy architecture of IPF. Areas with intensive ferroptotic activity exhibit high lipid peroxidation and iron accumulation, often colocalizing with fibroblastic foci, suggesting spatial coupling between ferroptosis-induced epithelial injury and fibroblast activation (126).

Importantly, these findings imply that ferroptosis inhibitors such as liproxstatin-1 may not exert uniform effects across fibroblast subsets. Instead, they may preferentially suppress the expansion of profibrotic fibroblast lineages, offering a potential avenue for targeted antifibrotic therapy tailored to fibroblast heterogeneity.

### 4.4 ECM production and fibrotic niche reinforcement

Ferroptosis-induced oxidative stress significantly influences ECM remodeling, a hallmark of IPF. The oxidative milleu enhances ECM stability and stiffness through the upregulation of enzymes like lysyl oxidases (LOX and LOXL2), which catalyzes the crosslinking of collagen and elastin fibers, increasing EMC tensile strength (127, 128). This stiffened ECM not only impairs lung compliance but also serves as a mechanical stimulus that activates fibroblasts through mechanotransduction pathways (117).

One consequence of increased ECM stiffness is suppression of miR-29, a key negative regulator the expression of stromal genes, resulting to increased translation of ECM components and further matrix deposition. Simultaneously, mechanical cues activates the Hippo pathway effector Yes-associated protein 1 (YAP), which translocates to the nucleus, where it promotes the transcription of profibrotic genes, reinforcing ECM deposition and stiffness (129). Additionally, mesenchymal progenitor cells acquire a mechanical memory mediated by miR-21, enabling them to maintain a fibrogenic phenotype even after the initial stiffening stimulus has subsided (130).

Ferroptosis of AECs exacerbates these effects by generating localized oxidative stress, which contributes to ECM stiffening and creates a fibrotic niche that fosters myofibroblast persistence. This self-reinforcing loop—linking ferroptosis, oxidative ECM remodeling, and fibroblast activation—underscores the central role of ferroptosis in sustaining chronic fibrotic remodeling in IPF.

Given the heterogeneity of fibroblast populations, such ECM-driven reinforcement of fibrosis may disproportionately affect specific fibroblast subsets that are more prone to profibrotic activation, further emphasizing the importance of context-dependent therapeutic strategies that consider fibroblast diversity and their variable sensitivity to ferroptotic stress.

## 5 Ferroptosis as therapeutic target for IPF

Given its pivotal role in alveolar epithelial injury and its contribution to inflammation and fibrotic remodeling, ferroptosis has emerged as a compelling therapeutic target in IPF. Multiple strategies have been proposed to modulate ferroptosis, targeting different components of its regulatory network and downstream signaling cascades. These approaches can be broadly categorized according to their target, including regulators of oxidative stress, lipid peroxidation, iron dysregulation, DAMP-mediated inflammation, profibrotic cytokines, and ECM remodeling (**Table 1**).

**Table 1:** Summary of ferroptosis inhibitors and their efficacy in IPF models.

Therapeutic Agent	Mechanism of Action	Efficacy in IPF Models	References
Deferoxamine (DFO)	Iron chelator; reduces Fe^2+^-driven lipid peroxidation via Fenton reaction inhibition	Reduced iron accumulation, decreased ferroptosis markers (e.g., 4-HNE), preserved epithelial integrity	(126, 131, 132)
Deferiprone (DFP)	Iron chelator; similar mechanism as DFO	Protective in oxidative stress-related disorders; potential to mitigate ferroptosis in pulmonary settings	(133–135)
Dihydroquercetin	Inhibits NCOA4-mediated ferritinophagy	Reduces intracellular iron release, alleviates AEC ferroptosis in silica-induced pulmonary fibrosis	(136)
N-Acetylcysteine (NAC)	GSH precursor; enhances antioxidant capacity	Limited efficacy in clinical trials (e.g., PANTHER-IPF); mechanistically relevant for ferroptosis inhibition	(137–139)
MitoTEMPO	Mitochondria-targeted antioxidant; scavenges mtROS	Decreased lipid peroxidation and fibrosis; preserves mitochondrial and epithelial integrity	(140)
MitoQ	Mitochondrial ROS scavenger; inhibits TGF-β/NOX4 and PDGF/ROCK pathways	Protective in lung injury; reduces cytokine secretion and fibrosis	(141, 142)
Ferrostatin-1 (Fer-1)	Lipid ROS scavenger; stabilizes GPX4	Reduces collagen deposition and TGF-β signaling; preserves epithelial structure	(124, 126, 143)
Liproxstatin-1 (Lip-1)	Inhibits lipid peroxidation via GPX4 activation	Similar effects as Fer-1; also activates NRF2 to amplify antioxidant defense	(124, 126, 143)
Sulforaphane	Natural NRF2 activator; boosts GPX4 and antioxidant enzyme expression	Decreased 4-HNE and fibrosis; restores redox balance in lung tissue	(144, 145)
Setanaxib (GKT137831)	Dual NOX1/4 inhibitor; blocks ROS generation	Shown to reduce fibrosis in preclinical models; Phase II trial in IPF patients recently completed	(146)
Rosiglitazone	ACSL4 inhibitor; reduces PUFA incorporation into membranes	Preserves GSH and GPX4 levels; protects against lipid peroxidation and fibrosis	(25)
AS-252424	Selective ACSL4 inhibitor	Anti-ferroptotic and anti-fibrotic effects in preclinical models	(147)

### 5.1 Regulators of ferroptosis core machinery

#### 5.1.1 Iron chelation and ROS management

Ferroptosis is an iron-dependent, regulated form of cell death characterized by iron-driven lipid peroxidation. In IPF, iron overload in alveolar space exacerbates oxidative stress and sensitizes AECs to ferroptosis by promoting Fenton chemistry and lipid peroxidation (148, 149). This process is especially damaging in the context of chronic lung injury, where sustained epithelial death and release of DAMPs propagate inflammation and fibrotic remodeling.

Supporting the translational relevance of these mechanisms, a clinical study by Puxeddu et al. (131) demonstrated the presence of iron-laden macrophages and elevated ferritin expression in lung tissue from IPF patients (131). These findings provide direct evidence of local iron accumulation and oxidative stress in the human fibrotic lung, reinforcing the pathophysiological rationale for targeting iron in therapeutic strategies.

Accordingly, strategies that reduce the bioavailable iron pool within the lung microenvironment have shown promise in mitigating ferroptosis and preserving epithelial integrity. Clinically approved iron chelators such as deferoxamine (DFO) and deferiprone (DFP), which are used to treat systemic iron overload, have demonstrated potential antifibrotic effects through inhibition of ferroptosis. In preclinical model of lung injury, DFO reduced iron accumulation, decreased ferroptosis markers such as 4-HNE, and preserve epithelial integrity, ultimately leading to attenuated fibrotic remodeling (126, 132, 133). Similarly, DFP has shown protective effects in models of neurodegenerative and retinal disorders by alleviating oxidative stress caused by excess iron (134–136). These chelators act by binding free Fe^2+^, thereby inhibiting the Fenton reactions and thus blocking a key driver of ferroptotic cell death.

Beyond chelation, modulation of ferritinophagy—a process that release stored iron from ferritin—offer another promising approach to reduce intracellular iron level (137). NCOA4—mediated ferritinophagy increases the available iron pool by releasing stored iron from ferritin complexes, thereby promotes ferroptosis under oxidative conditions. As discussed in Section 2.1, pharmacologic inhibition of ferritinophage, such as with dihydroquercetin, has been shown to ameliorate silica-induced pulmonary fibrosis by protecting AECs from ferroptosis (137). Thus, targeting NCOA4 could provide a more complementary, cell-intrinsic strategy for limiting ferroptotic injury. When combined with iron chelation, NCOA4 inhibition could yield a more robust and sustained suppression of ferroptosis, reinforcing epithelial resilience in the fibrotic lung.

#### 5.1.2 Antioxidant defense modulators

Oxidative stress is a key upstream driver of ferroptosis in IPF, making antioxidant defense modulators as attractive therapeutic candidates. By replenish glutathione (GSH), scavenging ROS, or enhancing endogenous antioxidant enzymes, these agents can interrupt the lipid peroxidation cascade and protect AECs from ferroptotic death.

N−Acetylcysteine (NAC), a precursor for GSH synthesis, has long been explored for oxidative stress mitigation. However, large randomized controlled trials, including the PANTHER−IPF study, revealed limited clinical efficacy of NAC alone therapy in slowing disease progression or improving survival in unselected IPF cohort (138, 139, 150). Although NAC’s ability to restore intracellular GSH and modulate ferroptotic markers remains mechanistically relevant, its limited benefit in clinical practice suggests a need to prioritize more targeted and potent antioxidants in future therapeutic design.

A growing body of evidence highlights the pivotal role of mitochondrial ROS (mtROS) in amplifying lipid peroxidation and sensitizing cells to ferroptosis (140). Accordingly, mitochondria-targeted antioxidants have gained traction. MitoTEMPO, a superoxide dismutase mimetic designed to localize within mitochondria, effectively scavenges mtROS and preserves mitochondrial integrity, significantly reducing ferroptosis and fibrosis exacerbation in experimental models (141). Similarly, MitoQ, a mitochondria-directed ubiquinone derivative, demonstrates protective effects in lung injury models by suppressing mtROS, decreasing proinflammatory cytokine secretion, and inhibiting TGF-β/NOX4 and PDGF/ROCK signaling pathways (142, 143). These agents exemplify organelle-specific antioxidant defense strategies that not only block ferroptosis but also interfere with pro-fibrotic signaling cascades.

Ferroptosis-specific inhibitors offer another line of therapeutic intervention. Small molecules such as ferrostatin-1 (Fer-1) and liproxstatin-1 (Lip-1) stabilize the activity of GPX4, the core enzyme that detoxifies lipid peroxides and suppresses ferroptotic cell death. In bleomycin-induced lung fibrosis models, Fer-1 and Lip-1 preserve epithelial structure, reduce collagen deposition, and attenuate TGF-β signaling. These agents also activate the NRF2 pathway, further amplifying antioxidant gene expression (124, 126, 144). While these inhibitors remain at the preclinical stage, they continue to inform the mechanistic underpinnings of ferroptosis inhibition and serve as a proof of concept for targeting lipid peroxidation in IPF.

The NRF2 transcription factor orchestrates expression of GPX4, SLC7A11, and a suite of antioxidant enzymes.

Central to the antioxidant response is the NRF2–GPX4 signaling axis. NRF2 transcriptionally activates genes encoding antioxidant enzymes such as GPX4, SLC7A11, and others involved in GSH metabolism (7, 145). Pharmacological NRF2 activators have demonstrated robust protective effects in preclinical models by reducing 4-HNE, restoring redox homeostasis, and limiting AEC ferroptosis (146). Sulforaphane, a natural NRF2 agonist, has shown antifibrotic activity in lung models and may be a candidate for further development (145). Newer synthetic NRF2 activators with improved bioavailability are currently under preclinical investigation, with some poised for translational studies.

Translating antioxidant modulation into clinical application has proven challenging, but emerging agents are bridging this gap. Setanaxib (GKT137831) is a first-in-class dual inhibitor of NOX1/4, two isoforms of NADPH oxidase that generate ROS and contribute to ferroptosis-associated tissue injury. Setanaxib has shown antifibrotic efficacy in preclinical models of liver, kidney, and lung fibrosis (151). It is the first NADPH oxidase inhibitor to enter clinical trials for IPF. A phase II randomized, placebo-controlled, multicenter trial (NCT03865927) was recently completed to evaluate Setanaxib in ambulatory patients with IPF. Although results have not yet been posted, preliminary investigator updates suggest that Setanaxib may significantly reduce pulmonary injury and disease progression by limiting NOX-derived ROS and interfering with ferroptosis-associated pathways. This agent represents one of the most clinically advanced antioxidant-based therapies aimed at IPF.

Together, these antioxidant modulators offer multiple points of intervention along the ferroptotic cascade. While clinical translation has been challenging, ongoing trials of NRF2 activators and novel formulations may yet unlock significant benefits for IPF patients.

#### 5.1.3 Targeting lipid metabolism

Lipid peroxidation is a hallmark of ferroptosis and a major contributor to alveolar epithelial injury in IPF (57)​. Therapeutic strategies that disrupt the enzymatic or non-enzymatic formation of lipid hydroperoxides may directly suppress ferroptotic cell death and attenuate the downstream fibrotic cascade. Among these, ACSL4 plays a key role by facilitating the incorporation of PUFAs into membrane phospholipids, thereby sensitizing cells to peroxidation and ferroptosis (147).

Inhibition of ACSL4 has shown therapeutic promise in preclinical models of fibrosis. Rosiglitazone, a thiazolidinedione initially developed as a PPARγ agonist, was found to attenuate ischemia–reperfusion–induced lung injury by suppressing ACSL4 activity. This intervention was associated with reduced lipid peroxidation and preserved intracellular levels of glutathione (GSH) and glutathione peroxidase 4 (GPX4), two essential components of cellular defense against ferroptosis (25)​. More recently, a selective small-molecule ACSL4 inhibitor, AS-252424, exhibited both anti-ferroptotic and anti-fibrotic effects in experimental settings, further underscoring the therapeutic potential of directly modulating lipid metabolism in the context of IPF (152).

Although the clinical translation of ACSL4 inhibitors remains in its early stages, these preclinical findings highlight the importance role of lipid remodeling in ferroptosis-driven fibrotic progression and suggest that future therapies strategies may benefit from targeting this metabolic axis.

### 5.2 Inflammatory signaling and immune activation

Regulated cell death pathways, including non−lytic apoptosis and lytic forms such as necroptosis, pyroptosis, and ferroptosis, release DAMPs that trigger sterile inflammation in the lung (153, 154). Key DAMPs such as HMGB1 and ATP activate PRRs, including TLR4 and receptor for advanced glycation end products (RAGE), on macrophages, neutrophils, and fibroblasts. This engagement leads to NF−κB–dependent upregulation of pro−inflammatory cytokines (TNF−α, IL−1β, IL−6) and chemokines, perpetuating tissue injury and fibrogenesis (155, 156).

Small−molecule inhibitors of TLR4 signaling have shown preclinical efficacy in lung injury models. TAK−242 (Resatorvid), a selective TLR4 inhibitor, suppresses NF−κB activation, reduces cytokine releases, and mitigates alveolar damage in models of acute lung injury (157). Eritoran (E5564), an MD2–TLR4 antagonist, was well tolerated in a Phase II sepsis trial (NCT00046072) and demonstrated trends toward lower mortality, suggesting translational potential in IPF (158).

Targeting RAGE signaling may also offer therapeutic benefit. FPS−ZM1, a blood−brain−barrier−permeable RAGE inhibitor, significantly attenuates HMGB1-mediated pulmonary inflammation and tissue injury in murine emphysema models, illustrating proof of concept for its use in fibrotic lung disease (159).

Direct inhibition of NF−κB signaling can dampen cytokine storms and fibrotic remodeling. BAY 11−7082 irreversibly inhibits IκB kinase, suppressing TNF−α–induced IκBα phosphorylation and reducing pro−coagulant and inflammatory markers in alveolar epithelial cells (160). Dimethyl fumarate (DMF), an FDA−approved NRF2 agonist for multiple sclerosis, indirectly inhibits NF−κB and has demonstrated antifibrotic effects by restoring redox homeostasis in aged IPF models (161).

The chemokine CCL2 (MCP−1) drives monocyte recruitment and differentiation into profibrotic monocyte−derived alveolar macrophages (Mo−AMs). Carlumab, an anti−CCL2 monoclonal antibody, demonstrated safety in Phase 1b oncology trials, though with limited monotherapy activity; its repurposing for IPF is under investigation (162). MLN1202, an anti−CCR2 antibody, reduced macrophage infiltration in Phase II rheumatoid arthritis studies, highlighting its potential to disrupt monocyte−macrophage-mediated fibrosis (163).

### 5.3 Cytokines and macrophage polarization

Ferroptosis-driven inflammation promotes the polarization of macrophages toward an M2-like, pro-fibrotic phenotype, largely mediated by cytokines such as TGF-β and IL-10 (164). Targeting these cytokines or their downstream signaling pathways offers a strategy to disrupt the self-perpetuating loop linking ferroptotic cell death to fibroblast activation and ECM deposition.

TGF−β and IL−10 are central to M2 macrophage polarization in the fibrotic lung. TGF−β, elevated downstream of ferroptosis, not only stimulates collagen production by fibroblasts but also induces the expression of M2 markers including arginase−1 and the mannose receptor (CD26) in macrophages, cementing their M2 identity. Therapeutic approaches targeting TGF-β signaling have shown partial success in clinical trials for IPF. Fresolimumab (GC1008), a pan−TGF−β neutralizing monoclonal antibody, completed a Phase I trial (NCT00125385) in IPF patients, demonstrating good tolerability and biomarker reductions suggestive of anti−fibrotic activity (165). Pirfenidone, an approved IPF antifibrotic, indirectly inhibits TGF−β1–mediated epithelial-mesenchymal transitions by suppressing MUC1−CT phosphorylation and β−catenin signaling, reducing α−SMA and collagen expression in preclinical models (166). Nintedanib, another approved IPF therapy, targets multiple tyrosine kinases downstream of PDGF and FGF, which intersect with TGF−β signaling to limit myofibroblast proliferation. In a Phase II trial, nintedanib effectively slowed lung function decline and reduced the frequence of acute exacerbation in patients with IPF (167).

IL−10, another key cytokine released by ferroptotic AECs, reinforces M2 polarization and tissue−repair functions in macrophages. Although IL−10 blockade remains preclinical in fibrosis, studies in murine models demonstrate that anti−IL−10 antibodies or genetic deletion of IL−10 signaling shifts macrophages toward a less fibrogenic profile and reduces collagen accumulation (168).

IL−6 lies at the interaction of inflammation and fibrosis. By promoting STAT3 activation, IL−6 fosters macrophage survival and M2 marker expression, while also driving fibroblast–myofibroblast transition. Tocilizumab, an IL−6 receptor antagonist approved for systemic sclerosis–associated interstitial lung disease (SSc−ILD), has shown promising results in preserving lung function and reducing M2−associated biomarkers in clinical studies (169, 170). While its role in IPF remains under investigation, the findings from SSc−ILD suggest that IL−6 blockade may help rebalance macrophage phenotypes and limit fibrotic progression.

Collectively, targeting cytokine and chemokine involved in ferroptosis-induced macrophage polarization presents a compelling strategy to reprogram the fibrotic immune environment in IPF. Future trials should explore these interventions—ideally in combination with ferroptosis inhibitors—to achieve synergistic suppression of epithelial injury and pro−fibrotic immune activation.

### 5.4 Fibroblast activation and ECM remodeling

Ferroptosis−associated inflammation activates myofibroblasts, which secrete matricellular proteins such as periostin and fibronectin. These proteins accumulate in the lung interstitium, and drive matrix stiffening and epithelial stress (129). This remodeled, rigid extracellular matrix drives nuclear translocation of YAP and TAZ, the Hippo pathway effectors, which in turn induce glycolytic and glutaminolytic genes that support myofibroblast survival and ECM production (171). Simultaneously, oxidative modifications of chromatin—such as histone H3K27 demethylation—enable YAP/TAZ to access fibrogenic promoters, sustaining an activated fibroblast phenotype (172). Therapeutic strategies targeting this axis, such as verteporfin to agents that modulate redox-sensitive epigenetic marks, hold promise for reversing fibroblast activation in IPF.

Several ECM-targeted therapies have advanced to clinical evaluation. Pamrevlumab (FG−3019), a fully human monoclonal antibody against connective tissue growth factor (CTGF/CCN2), was tested in the Phase II PRAISE trial (NCT01890265). In 103 IPF patients, pamrevlumab reduced the decline in forced vital capacity (FVC) by 60.3% compared to placebo over 48 weeks (–2.9% *vs* –7.2%; between−group difference of 4.3%, p=0.033) and halved the rate of disease progression (10.0% *vs* 31.4%), with a favorable safety profile (173).

PRM−151, a recombinant form of human pentraxin−2, also showed potential in IPF. In a Phase II randomized, placebo−controlled study (NCT02550873) of 111 IPF patients, PRM−151 slowed FVC decline over 28 weeks and preserved 6−minute walk distance, with good tolerability (174). A 76-week open−label extension confirmed sustained effects on lung function, consistent with its role in inhibiting fibrocyte differentiation and ECM production (175). However, the subsequent Phase III STARSCAPE trial (NCT04552899) was terminated early for lack of efficacy, though prior data still validate pentraxin−2 as a regulator of fibroblast activity (175).

Admilparant (BMS−986278), an oral lysophosphatidic acid (LPA) receptor antagonist, has been evaluated in a Phase II randomized trial (NCT04308681) in IPF and progressive pulmonary fibrosis. A 26-week course of 60 mg twice daily led to a 69% relative reduction in the rate of percent predicted FVC decline versus placebo (treatment difference 2.9%; 95% CI 0.4-5.5), with a favorable safety profile (176). These encouraging results support its progression to Phase III evaluation.

Emerging evidence indicates that ferroptosis−associated lipid peroxidation and iron overload not only contribute to ECM stiffening but also reprogram fibroblast metabolism and epigenetics to keep cells into an activated state. Disrupting key steps in ECM remodeling, including matricellular protein deposition, collagen crosslinking, and mechanotransduction, represents a promising strategy to halt fibrosis progression. When combined with upstream ferroptosis inhibitors, this multipronged strategy holds promise for preserving lung architecture and halting IPF progression.

## 6 Conclusion and perspective

Ferroptosis, an iron−dependent, lipid peroxidation–driven form of regulated cell death, has emerged as a key pathophysiological mechanism in IPF, linking alveolar epithelial injury to persistent inflammation, macrophage activation, and fibrotic remodeling of the lung parenchyma (7). This review integrates core mechanisms—dysregulated iron metabolism, compromised antioxidant defenses, enzymatic and non−enzymatic lipid peroxidation, and mechanotransduction via YAP/TAZ—with cellular crosstalk among epithelial cells, immune populations, and fibroblasts. We also discuss therapeutic strategies ranging from iron chelators to inhibitors of mechanosensitive pathway (6). Together, these insights offer a systems-level view of ferroptosis as both a driver and a modifiable target in IPF pathogenesis (177).

However, several major challenges must be addressed to enable clinical translation of ferroptosis-targeted therapies. Iron chelators such as deferoxamine and deferiprone, while effective in mitigating epithelial ferroptotic injury in preclinical models, carry the risk of systemic iron depletion and may exacerbate anemia-a condition already prevalent among IPF patients due to chronic inflammation and reduced erythropoiesis (178). Similarly, antioxidant therapies like NAC, while aim to restore intracellular glutathione and reduce ROS-dedicated lipid peroxidation, have shown inconsistent efficacy in IPF. Notably, PANTHER−IPF study reported no improvement in lung function and even raised safety concerns when NAC was combined with immunosuppressants (139, 150). These findings highlight the complex and context-dependent role of ROS in IPF pathogenesis, where both insufficient and excessive ROS can be detrimental. Direct ferroptosis inhibitors, including ferrostatin−1 and liproxstatin−1, exhibit potent *in vitro* protection but suffer from poor oral bioavailability, uncertain pharmacokinetics, and potential off−target toxicity, complicating their clinical development (177). Enzyme−targeted approaches, such as ACSL4 inhibition, remain confined to early preclinical stages, with no candidates yet advancing to human trials.

Beyond these individual drug challenges, broader translational barriers exist: achieving sufficient distribution in fibrotic lung tissue, avoiding disruption of physiological redox signaling, and minimizing toxicity during long-term administration (177). Emerging delivery platforms such as nanoparticle−based delivery systems and inhalable formulations offer promising avenues to enhance lung targeting and reduce systemic exposure, but their efficacy and safety in IPF-specific contexts remain to be rigorously validated (179).

Future research efforts should prioritize the identification of ferroptosis-specific biomarker to improve clinical translation. These may include genetic, proteomic, or metabolomic indicators that not only confirm the presence of ferroptotic activity but also help stratify patients likely to benefit from targeted interventions. Such biomarkers would be invaluable for selecting appropriate candidates for clinical trials and for monitoring treatment responses in real time (180).

Another important direction is the development of combination therapies that simultaneously target ferroptosis and other pro-fibrotic mechanisms. For instance, co-targeting ferroptotic pathways along with TGF-β/Smad signaling or YAP/TAZ-driven mechanotransduction may produce synergistic effects that surpass those of monotherapies. Achieving this will require a deeper mechanistic understanding of how ferroptosis interacts with these parallel pathways in different lung cell types and microenvironments (7).

Advances in spatial multi-omics technologies hold promise for resolving the spatial heterogeneity of ferroptotic activity within the fibrotic lung. These approaches can identify distinct cell populations and regions with heightened ferroptotic stress, enabling the design of more precise and localized therapies. By integrating spatial transcriptomics, proteomics, and metabolomics, researchers can develop targeted interventions based on the cellular context and disease stage (181).

Recent discoveries also highlight the role of RNA editing and epigenetic regulation in ferroptosis. In particular, ADAR1−mediated adenosine−to−inosine RNA editing has been shown to modulate ferroptosis-regulated microRNAs such as let−7d, which intersect with both TGF−β signaling and oxidative stress pathways; restoring normal ADAR1 function could mitigate ferroptosis−driven miRNA dysregulation and stabilize both epithelial and fibroblast phenotypes (182).

In conclusion, ferroptosis represents both a mechanistic cornerstone and a therapeutic target in IPF. By addressing these key knowledge gaps—ranging from biomarker discovery to epigenetic control—future studies can establish the foundation for ferroptosis-targeted IPF therapies (183). Such advances hold the potential to transform how we diagnose, monitor, and treat this devastating and currently incurable disease.
